# Bloodstream infections in Cameroon: a systematic review and meta-analysis

**DOI:** 10.1016/j.nmni.2025.101654

**Published:** 2025-10-14

**Authors:** Moise Matakone, Sen Claudine Henriette Ngomtcho, Patrice Landry Koudoum, Isaac Dah, Ravalona Jessica Zemtsa, Michel Noubom

**Affiliations:** a Department of Microbiology, Haematology and Immunology, Faculty of Medicine and Pharmaceutical Sciences, University of Dschang, Dschang, Cameroon; b The National Veterinary Laboratory (LANAVET), Yaoundé, Cameroon; c Molecular Biology Unit, National Public Health Laboratory, Yaoundé, Cameroon; d Genomic Surveillance Study Group, Ministry of Public Health, Yaoundé, Cameroon; e School of Veterinary Medicine and Science, University of Ngaoundéré, Ngaoundéré, Cameroon; f Department of Biochemistry, Faculty of Medicine and Biomedical Sciences, University of Yaoundé I, Yaoundé, Cameroon; g Annex Regional Hospital of Dschang (ARHD), Dschang, Cameroon

**Keywords:** Bloodstream infections, *Escherichia coli*, *Klebsiella*, *Staphylococcus aureus*, Antimicrobial resistance, Priority pathogens

## Abstract

**Background:**

Bloodstream infections (BSIs) are significantly associated with morbidity and mortality worldwide, and particularly in low-resource settings. We determined the prevalence of BSIs, profile, and antimicrobial resistance of the causing bacteria in Cameroon.

**Methods:**

PubMed, Google Scholar and ScienceDirect databases were searched to identify relevant studies. The random or fixed effect model was used depending on the level of heterogeneity among studies for pooling estimates after the variance was stabilised through the Freeman-Tukey double arcsine transformation. Begg's and Egger's tests were used to quantify publication bias. The protocol was registered in PROSPERO (CRD42023482760).

**Results:**

The analysis of 5680 blood cultures from ten studies revealed an overall bacterial BSI prevalence of 25.2 % (95 % CI: 18.1–33.2 %) and 17.2 % (11.6–23.7 %) when the potential contaminants were removed. The leading BSI-causing bacteria were *Escherichia coli*, *Klebsiella* species, and *Staphylococcus aureus*. We found significant resistance to clinically relevant antibiotics, particularly to extended-spectrum cephalosporins in *E. coli*, *Klebsiella* species and *Acinetobacter* species, whereas resistance rates were lowest against carbapenems. Staphylococci displayed resistance rates above 30 % to all tested antibiotics. WHO bacterial priority pathogens occupied a greater proportion of the overall BSI-driven bacteria.

**Conclusion:**

This study reports a substantial prevalence of BSI and resistance to the commonly used antibiotics. It also points out the lack of compliance with international guidelines in microbiological analysis. This highlights the need to empower laboratories’ capacities and conduct BSI surveillance across the country to develop more targeted strategies for the prevention, management and treatment of BSIs.

## Highlights

• Analysis of 5680 blood cultures revealed a 25.2 % prevalence of bacterial bloodstream infections in Cameroon, primarily caused by *Escherichia coli*, *Klebsiella* spp., and *Staphylococcus aureus.*
• Significant resistance to extended-spectrum cephalosporins was observed in *Escherichia coli*, *Klebsiella* spp., and *Acinetobacter* spp. Staphylococci displayed resistance rates of more than 30 % to all tested antibiotics.
• WHO priority pathogens, including carbapenem-resistant *Acinetobacter*, third-generation cephalosporin-resistant *Enterobacterales*, carbapenem-resistant *Enterobacterales*, fluoroquinolone-resistant *Salmonella* and methicillin-resistant *Staphylococcus aureus,* occupied a greater proportion of the overall bacteria causing bloodstream infections.

## 1 Introduction

Bloodstream infection (BSI) is the presence of viable microorganisms in the bloodstream of patients with systemic signs and symptoms of infection, and the positivity of one or more blood cultures [1,2]. BSIs are significantly associated with morbidity and mortality in both developed and developing countries. In 2019, BSIs were associated with 2.91 (1.74–4.53) million deaths worldwide [3]. The estimated BSI burden reported in 2013 from population-based studies in North America and Europe ranged between 113 and 204 (incidence) and between 20 and 38 (mortality) per 100,000 person-years, and ranked among the top seven causes of death in all included countries [4]. Data on BSI burdens from low-resource settings, particularly in Africa, are scarce. Nevertheless, a systematic review and meta-analysis reported a median prevalence of community-onset BSI among hospitalised patients in Africa and Asia of 12.5 % (3164 of 29,022) [5]. In sub-Saharan Africa (SSA), Melariri and colleagues reported a pooled prevalence of 36.8 % (2671 of 4500 cases) of hospital-acquired BSIs, even though Central Africa was the least represented SSA subregion in the study [6]. In Cameroon, a global view of BSI is not yet well established.

The bacterial pathogens leading BSIs vary from one region to another and depend on the origin (community-acquired, hospital-acquired or healthcare-associated-BSI)*. Escherichia coli*, *Klebsiella pneumoniae*, *Staphylococcus aureus, Streptococcus pneumoniae, Pseudomonas aeruginosa,* and coagulase-negative staphylococci are the most commonly reported bacteria worldwide [3,5,[7], [8], [9]]. According to the origin, some studies have associated *E. coli* and streptococci with community-acquired BSIs, *Pseudomonas aeruginosa* and staphylococci with healthcare BSIs, and *Acinetobacter baumannii* with hospital-acquired BSIs [7,10]. While gram-negative bacilli such as *E. coli*, *Klebsiella pneumoniae* and *Pseudomonas aeruginosa* have been highly reported from studies conducted in high-income countries, *Salmonella enterica* instead seems to be a typical BSI pathogen for low-resource settings [7], and mainly involved in community-acquired BSIs [5,8]. The leading cause of fatal BSIs was attributed to *Staphylococcus aureus,* with 299,000 deaths [0,0–166,166–485] reported in 2019 [3]. The spectrum of BSI organisms in Cameroon includes *E. coli*, *Klebsiella* species, *Staphylococcus aureus* and coagulase-negative staphylococci—against all expectations, *Salmonella* was rarely recorded in the majority of studies [[11], [12], [13], [14], [15], [16], [17], [18], [19], [20]]. This variation among BSI pathogens across regions highlights the importance of establishing local bacteriological profiles for each setting and country.

As with all medical emergencies, BSIs require early intervention, which is commonly an empirical antimicrobial therapy—blood culture, the gold-standard technique for identifying organisms causing BSI and choosing appropriate antibiotics, requires a long period of time to perform [21]. Early and adequate antibiotic administration within the first 24 h of admission was associated with reduced mortality and improved patient outcomes [[21], [22], [23]]. It should be noted, therefore, that early empirical antimicrobial therapy can also be inactive—the treatment has no intrinsic activity against the presumed microorganism [22]. Thus, the empirical antimicrobial therapy choice should consider several parameters, including but not limited to the documented local BSI epidemiology and antimicrobial resistance (AMR) status, particularly in healthcare settings with endemic multidrug resistance and/or the patient's recent exposure to antimicrobials [21]. Despite the scarcity of BSI data in Cameroon, summarising the available data is important for providing context-specific epidemiology to direct future BSI prevention and management efforts. This review estimates the pooled prevalence of culture-confirmed bacterial BSIs, the distribution of causing bacteria and their AMR rates in Cameroon.

## 2 Methods

This review followed the Preferred Reporting Items for Systematic Review and Meta-Analysis (PRISMA) 2020 guidelines (**supplementary file S1,** checklist). The protocol was registered in PROSPERO (Ref: CRD42023482760). The measured outcomes are [1] the prevalence of culture-confirmed bacterial BSIs [2], the profile of BSI-causing bacteria and [3] the AMR rates and phenotypes of the most represented bacteria.

### 2.1 Search strategy and selection criteria

The searched databases included PubMed, Google Scholar and ScienceDirect, combined with a manual search of the reference list of relevant articles and grey literature. Three authors (MM, PLK, and RJZ) independently searched the literature. We targeted studies published between January 2010 and July 2025 with no language restriction using keywords paired with Boolean operators (**S2**). Articles were uploaded to EndNote software (version 20.6), and duplicates were removed. Two reviewers (MM and PLK) screened the titles and abstracts to determine whether specific inclusion criteria were satisfied. We included all studies reporting the prevalence of culture-confirmed bacterial BSIs, profile of the isolated bacteria and their AMR in Cameroon. Studies that do not allow the calculation of prevalence (e.g., case reports/series, case-control studies) or those that presented aggregated data with other clinical samples or where inconsistencies were noted were excluded. However, studies that reported only positive blood cultures with antimicrobial susceptibility testing (AST) results at the species/genus level were included in the meta-analysis of AMR rates. Authors of relevant articles were contacted when supplementary data or clarifications were needed.

### 2.2 Quality appraisal

The quality of the included studies was assessed using the eight-item Critical Appraisal Checklist of the Joanna Briggs Institute (JBI) for cross-sectional studies [24]. An item was awarded a point for “yes”, and the final score for each article was added and fell between zero and eight. A study was considered low risk if it scored ≥4 points. Disagreements on whether an article should be included were resolved in a group discussion.

### 2.3 Data extraction

Data from the included studies were independently extracted following the Microbiology Investigation Criteria for Reporting Objectively (MICRO) guidelines by two authors (MM and PLK) via a Microsoft Excel spreadsheet (version 2019). Extracted data included, but were not limited to, the laboratory work (specimen processing, organism identification, AST method), first author, publication year, region of study, study design, sample size, age of the study population, prevalence of BSIs, proportion of each bacterium causing BSIs, and AMR rates of these bacteria.

### 2.4 Data analysis

Bacterial BSI was defined as clinically suspected cases where blood culture yielded relevant bacterial growth. If a study didn't specify the number of positive cultures, we assumed that it was equal to the number of isolated pathogens and vice versa. For the estimation of the overall BSI prevalence, we included all the reported pathogens in each study, and then we excluded the potential contaminants, coagulase-negative staphylococci, for instance. However, these potential contaminants were considered pathogenic if the authors clearly explained how they handled them properly, or there was clinical evidence, or the study was conducted in immunocompromised patients (cancer, HIV patients, etc), except for retrospective studies (data from the laboratory registers), they were systematically considered as contaminants. We didn't reinterpret zone diameters or the minimal inhibitory concentration (MIC) values based on the contemporary guidelines. However, bacteria reported as resistant or intermediate to an antibiotic by the authors were categorised as resistant. Resistance phenotypes are defined as in Table 1.

**Table 1:** Definitions of resistance phenotypes recorded in this review.

Phenotypes	Definition/criteria^a^	Targeted bacteria
Third-generation cephalosporins resistance	Isolates phenotypically resistant to at least one of the tested third-generation cephalosporins	*Enterobacterales*
Extended spectrum β-lactamase (ESBL) producers	Isolates phenotypically confirmed by either the double disc synergy or combined discs tests (in the case of studies included in this review)	*Enterobacterales*
Fluoroquinolone resistance	Isolates phenotypically resistant to at least one of the fluoroquinolones	*Salmonella* species
Carbapenem resistance	Isolates phenotypically resistant to at least one of the carbapenems	*Acinetobacter* species and *Enterobacterales*
Methicillin-resistant Staphylococcus aureus (MRSA)	*Staphylococcus aureus* isolates resistant to cefoxitin or confirmed by other methods	*Staphylococcus aureus*

a When a study reported the antibiotic susceptibility testing results of multiple third-generation cephalosporins, carbapenems, or fluoroquinolones and isolate-level data were unavailable, we recorded the highest number of resistant isolates reported for any antibiotic within the targeted resistance phenotype. For studies where different subsets of isolates were tested against different antibiotics (i.e., varying denominators), we used cefotaxime as the reference for third-generation cephalosporin resistance, imipenem for carbapenem resistance and ciprofloxacin for fluoroquinolone resistance.

Begg's and Egger's tests were used to quantify publication bias, and a funnel plot was used to visualise the bias. When asymmetry is evident in the funnel plot with or without a significant Begg's and Egger's test, the trim and fill method is applied to adjust the bias and the bias-adjusted pooled prevalence is calculated. However, publication bias was not assessed for pooled estimates with fewer than ten included studies because these tests are not reliable and underpowered [25]. The Q test and *I*^*2*^ statistic were used to assess heterogeneity between studies. The *I*^*2*^ value of zero indicates true homogeneity, whereas values of 25 %, 50 % and 75 % were considered low, moderate and high heterogeneity, respectively. Before pooling prevalence, variances were stabilised with the Freeman-Tukey double arcsine transformation. For each study, we used the Clopper-Pearson exact method to calculate the 95 % confidence intervals (CIs). The random effects model was systematically employed to pool estimates because of the expected high between-study heterogeneity. However, when true homogeneity or low heterogeneity was noticed for an estimate, we recalculated the pooled estimate with the fixed effects model. A leave-out one study test (where one study is left out at a time, and the pooled prevalence is recalculated) was employed to assess the robustness or influence of a single study on the pooled prevalence. Finally, we conducted a subgroup analysis according to the age group of the study population, the city and site where the study was carried out and the study design to assess potential sources of heterogeneity. A p-value <0.05 was considered to indicate statistical significance. Statistical analysis and visualisation were performed using R software, version 4.4.1 [26].

## 3 Results

**Fig. 1:**
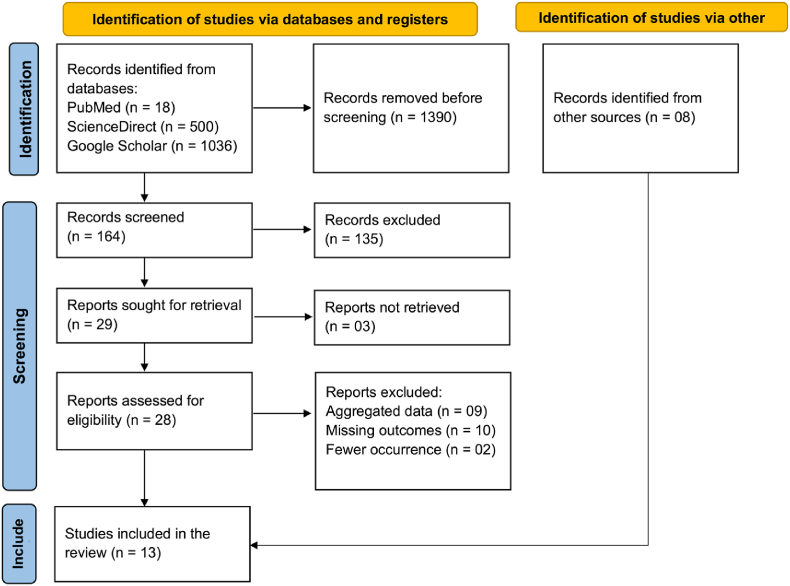
PRISMA flow chart of study selection process.

The online database searches in PubMed, ScienceDirect, and Google Scholar yielded 1554 articles; six studies were identified through a manual search, and two unpublished datasets were obtained directly from the authors. After duplicates and non-relevant articles were removed, 28 studies were assessed for eligibility, and 13 were included (Fig. 1). Six authors were contacted to request supplementary data, clarifications and the full text; two responded, but no data were provided.

Out of the 13 included studies, nine come from peer-reviewed journals [[11], [12], [13], [14], [15],^17^,^19^,^20^,^27^], one dataset from the World Health Organization (WHO) database [28], one dataset from a study conducted in 14 African countries and collected data from 16 laboratories in Cameroon [29] and two datasets of unpublished studies [30,31]. Data were collected primarily from tertiary category public hospitals from 2006 to 2023. Most of the studies were cross-sectional (n = 09) [[11], [12], [13], [14], [15],^19^,^20^,^27^,^31^], while two were retrospective (investigated blood culture results from laboratory registers) [17,30] (Table 2 and S3).

**Table 2:** Summary characteristics of the studies included in the meta-analysis.

Author	Study period	Study design	City	Sample size	Positive n
Chiabi et al., 2011 [12]	Nov 2008–May 2009	Cross-sectional	Yaoundé	189	26
Djuikoue et al., 2022 [13]	Aug 2019–Mar 2020	Cross-sectional	Yaoundé	300	130
Ebongue et al., 2014 [17]	2006–2011	Retrospective	Douala	2334	299
Kamga et al., 2011 [14]	Jan–Jun 2010	Cross-sectional	Yaoundé	396	112
Kemeze et al., 2016 [15]	Mar–Jun 2015	Cross-sectional	Douala	104	/
Titsamp et al., 2021 [11]	Aug–Oct 2017	Cross-sectional	Yaoundé	298	129
Yimtchi et al., 2023 [19]	Nov 2021–Mar 2022	Cross-sectional	Yaoundé	150	24
Zefack et al., 2020 [20]	Jan–Apr 2018	Cross-sectional	Yaoundé	255	65
Poundy et al., 2024 [27]	Jan and Sep 2021	Cross-sectional	Yaoundé	119	49
One Health Trust, 2025 [29]	2017–2019	/	/	/	/
Mimche [30]	Jan 2020–Apr 2023	Retrospective	Yaoundé	1365	216
Nguetsop [31]	Feb–Aug 2023	Cross-sectional	Douala	274	60
GLASS [28]	2020–2022	/	/	/	/

Collected blood volumes varied between 1-5 mL and 5–10 mL for children and adults, respectively. The manual blood culture was performed in most of the included studies, while two studies exclusively used the automated method (BACT/ALERT, BioMérieux) [27,31] and one study used both methods [30]. The number of blood cultures per participant was not stated in most of the included studies, and fewer used at least two sets. Organisms were identified using standard bacteriological techniques, including the examination of colony characteristics in petri dishes, Gram staining, catalase, oxidase, coagulase, and dnase tests, and confirmed with the API gallery and Slidex strepto plus reagent, depending on the bacterial group. Two studies utilised both the API gallery and Vitek 2 compact (BioMérieux) [27,30], an automated method, while one study employed only the automated method [31]. Most of the included studies used the disc diffusion method (Kirby-Bauer) for AST [[11], [12], [13], [14],^19^,^20^,^27^]. However, the determination of MIC was also performed using the Vitek 2 compact system [31] and the broth dilution method [17]. Two studies performed both Kirby-Bauer and MIC methods [29,30], while the study by Kemeze et al. did not describe the laboratory work. Most of the authors utilised the Comité de l’Antibiogramme de la Société Française de Microbiologie (CA-SFM) [11,13,17,19,27,30] guideline for the interpretation of the AST results; one study used instead the Clinical and Laboratory Standards Institute (CLSI) [20] guideline, one used both guidelines [29], and the other studies did not specify the reference used [12,14,15,28,31]. Additionally, a few studies reported the use of a control strain to validate the quality of their antibiotics [14,20,30]**(S3**).

The results of JBI critical appraisal show that overall included studies are low-risk of bias except one study whose score was low [15]. Since no contact of the corresponding author was provided in the manuscript to request further information, we decided to include it in the meta-analysis of one outcome (BSI-causing bacteria estimation) that we deemed well-reported. One author was requested supplementary data and clarifications, even though the score was acceptable; only clarifications were provided and was therefore included in the meta-analysis of two out of the three measured outcomes [27]. We did not apply a formal JBI appraisal to two studies (datasets) because the detailed methodological information was unavailable [[28], [29], [30]] (**S4**).

### 3.1 Prevalence of bacterial bloodstream infections in Cameroon

**Fig. 2:**
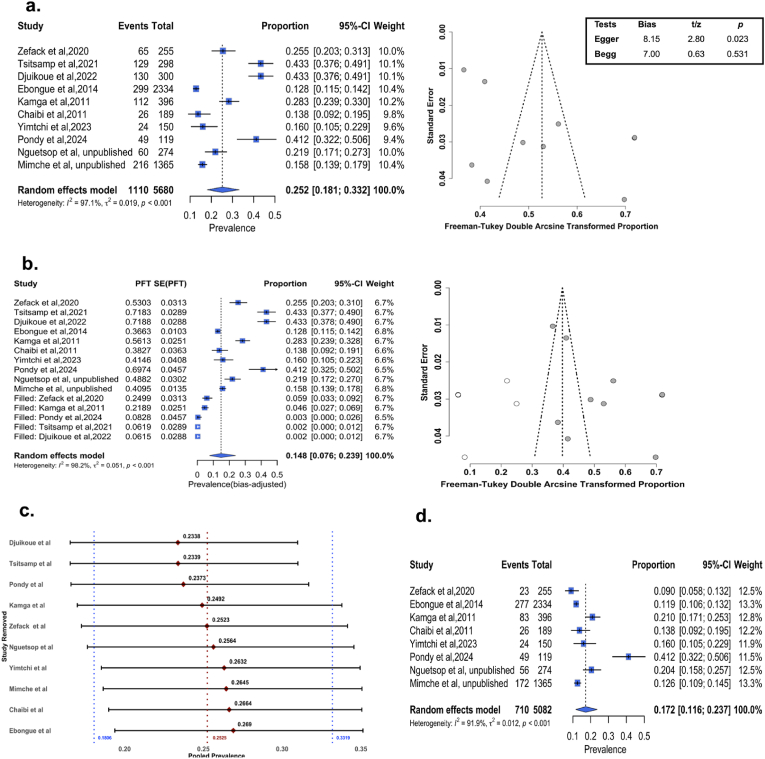
Prevalence of bacterial bloodstream infections in Cameroon. **(a)** From left to right, forest plot displaying BSI prevalence (squares represent prevalence and 95 % confidence interval per study and the diamond the overall prevalence and 95 % CI), funnel plot showing asymmetrical distribution of studies and Egger and Begg tests quantifying publication bias; **(b)** From left to right, bias-adjusted forest and funnel plots after the trim and fill test (empty circles represent the added studies and dark the original ones); **(c)** Sensitivity result showing a variation of prevalence of less than two percent from the overall pooled prevalence after one study is removed at a time. The red diamonds represent the new pooled prevalence and the 95 % CI after one study was removed; red and blue dot lines are the overall prevalence and 95 % CI, respectively. **(d)** Forest plot showing the prevalence of bacterial bloodstream infections after potential contaminants were removed.

Ten studies were included in the meta-analysis of BSI prevalence, investigating 5680 blood cultures, of which 1655 (29.14 %) were performed in children under 18 years old, and 119 were children with cancer. All these studies were conducted in two of the 10 regions of Cameroon and included hospitals from only two cities: Douala, the economic capital (45.92 %, number of blood cultures or isolates depending on the context (n) = 2,608, number of studies (k) = 02) [17,31] and Yaoundé, the political and administrative capital (54.08 %, n = 3,072, k = 08) [[11], [12], [13], [14],^19^,^20^,^27^,^30^]. Studies by Ebongue et al. [17], conducted at the general hospital of Douala, and by Mimche [30] at the central hospital of Yaoundé, comprised more than half of the participants included in the BSI meta-analysis. The BSI prevalence in included studies ranged from 12.8 % [17] to 43.4 % [11,13], with a high between-study heterogeneity observed (*I*^*2*^ = 97.1 %). Thus, the pooled prevalence from the random effect model was 25.2 % (95 % CI: 18.1–33.2 %, n = 5,680, k = 10) (Fig. 2a, S5). When the potential contaminants were removed from studies, we obtained a prevalence of 17.2 % (11.6–23.7 %, n = 5082, k = 08). Two studies were not included because they did not provide the full list of isolated bacteria [11,13] (Fig. 2d).

Investigation of publication bias through a visual inspection of the funnel plot showed asymmetry and the Egger regression test was also significant (bias estimate: 8.15, p = 0.023), suggesting possible publication bias, although the Begg's test was not significant (Fig. 2a). Since the number of included studies is at the threshold of the recommended number of studies for a reliable assessment of publication bias and given the high heterogeneity across studies, the asymmetry might also due to the between-study difference rather than publication bias. Nevertheless, we performed the trim and fill test to adjust the bias, and five studies were added, dropping the prevalence to 14.8 % (7.6–23.9 %, *I*^*2*^ = 98.2 %) (Fig. 2b).

The sensitivity results showed an overall variation of less than 2 % from the pooled prevalence—a minimum of 23.4 % when the study by Titsamp et al. or Djuikoue et al. was removed, and a maximum of 26.9 % when Ebongue et al. was removed. This suggests that the influence of a single study is not significant, and the pooled prevalence is likely stable (Fig. 2c–S6).

**Fig. 3:**
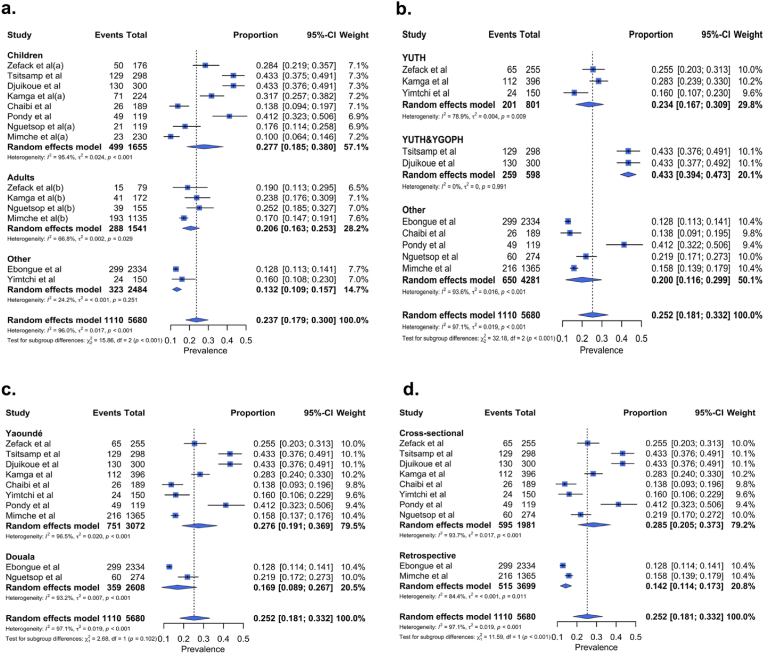
Forest plots of subgroup analysis. **(a)** The age subgroup, the children's group includes participants under 18 years old, and the other group comprises studies where data on participants' age were unavailable. **(b)** The study site subgroup refers to the hospital setting where data were collected and/or analysed. The other group includes the Yaoundé Central Hospital, Douala General Hospital, Douala Gynaeco-Obstetric and Paediatric Hospital, Laquintinie Hospital of Douala, and the Mother and Child Centre of the Chantal Biya Foundation. **YUTH:** Yaoundé University Teaching Hospital, **YGOPH**: Yaoundé Gynaeco-Obstetric and Paediatric Hospital.

The subgroup analysis revealed that the prevalence of BSI was more important among children (27.7 %, 18.5–30.0 %, n = 1,655, k = 08) compared to adults (20.6 %, 16.3–25.3 %, n = 1,541, k = 04), and a substantial drop of heterogeneity across the groups was noticed (Fig. 3a, S7). A similar result was observed in the study sites group, the BSI prevalence was two times higher in studies conducted at both the Yaoundé University Teaching Hospital (YUTH) and the Yaoundé Gynaeco-Obstetric and Paediatric Hospital (YGOPH) (43.3 %, 39.4–47.3 %, n = 598, k = 02) compared to those exclusively carried out at the YUTH (23.4 %, 16.7–30.9 %, n = 801, k = 03) or in other hospital settings (20.0 %, 18.1–33.2 %, n = 4,281, k = 05) (Fig. 3b). However, in the study design and study city subgroups, although prevalence varied across modalities within each subgroup, the heterogeneity remained elevated (Fig. 3c and d). These findings suggest that age and study sites might have contributed partially to the observed heterogeneity.

### 3.2 Bacterial aetiology of bloodstream infections

**Fig. 4:**
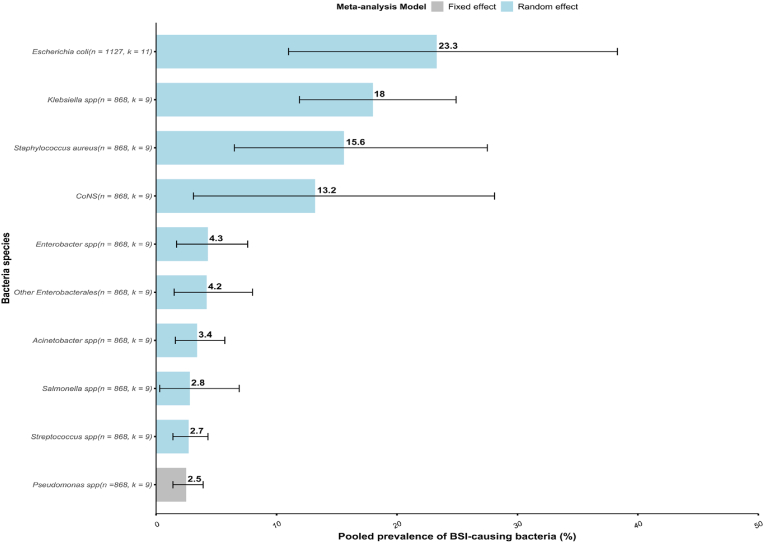
Pooled prevalence of bloodstream infection-causing bacteria. Among *Klebsiella* species, 138 out of 170 were *Klebsiella pneumoniae*; among *Salmonella* species 11 out of 53 were *Salmonella* Typhi; the other *Enterobacterales* includes *Citrobacter* species (n = 08), *Yersinia enterocolitica* (n = 02), *Proteus* species (n = 07), *Serratia* species (n = 03), *Providencia* species (n = 07), *Morganella morganii* (n = 01), *Pantoea* species (n = 04), *Kluyvera cryocrescens* (n = 01), *Escherichia hermannii* (n = 01), and unspecified *Enterobacterales* (n = 26); among *Acinetobacter* species, 11 out of 31 were *Acinetobacter baumannii*; among *Pseudomonas* species, 18 out of 29 were *Pseudomonas aeruginosa*; coagulase negative staphylococci includes *Staphylococcus epidermidis* (n = 47), *Staphylococcus saprophyticus* (n = 19), *Staphylococcus haemolyticus* (n = 12), *Staphylococcus simulans* (n = 01), *Staphylococcus gallinarum* (n = 01), *Staphylococcus xyloxus* (n = 02), *Staphylococcus hominis* (n = 03), *Staphylococcus lugdunensis* (n = 01), *Staphylococcus cohnii* (n = 01), *Staphylococcus* species (n = 02) and unspecified CoNS (n = 66).

The leading bacteria causing bloodstream infections in Cameroon were *Escherichia coli* 23.3 % (11.0–38.3 %, n = 1,127, k = 11), followed by *Klebsiella* species 18.0 % (11.9–24.9 %, n = 868, k = 09), and *Staphylococcus aureus* 15.6 % (6.5–27.5 %, n = 868, k = 09). The least reported bacteria included, but were not limited to, *Salmonella* species 2.8 % (0.3–6.9 %, n = 868, k = 09), *Streptococcus* species 2.7 % (1.4–4.3 %, n = 868, k = 09) and *Pseudomonas* species 2.5 % (1.4–3.9 %, n = 868, k = 09) (Fig. 4, sFig. 1 and S8-9).

### 3.3 Antimicrobial resistance of BSI-causing bacteria

**Fig. 5:**
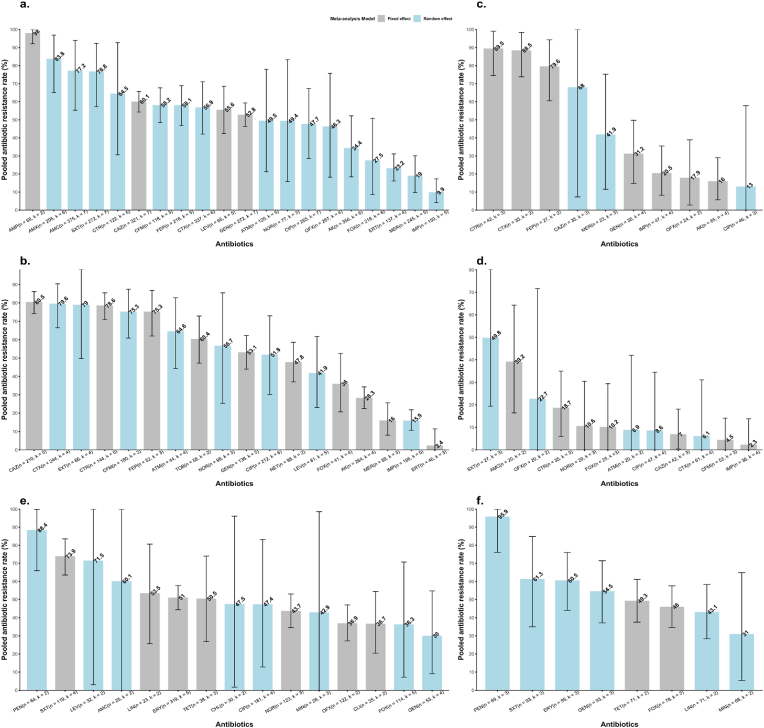
Pooled antibiotic resistance rates and 95 % confidence intervals of BSI-causing bacteria in Cameroon. n and k represent, respectively, the total number of tested isolates and the studies included. **(a)** AMR rate of *Escherichia coli* against twenty antibiotics. **(b)** AMR rate of *Klebsiella* species against eighteen antibiotics. **(c)***Acinetobacter* species. **(d)***Salmonella* species. **(e)***Staphylococcus aureus*. **(f)** Coagulase-negative *Staphylococcus.* Amikacin (AK), Amoxicillin (AMX), Amoxicillin + clavulanic acid (AMC), Ampicillin (AMP), Aztreonam (ATM), Cefepime (FEP), Cefixime (CFM), Cefotaxime (CTX), Cefoxitin (FOX), Ceftazidime (CAZ), Ceftriaxone (CTR), Chloramphenicol (CHL), Ciprofloxacin (CIP), Clindamycin (CLI), Cotrimoxazole (SXT), Ertapenem (ERT), Erythromycin (ERY), Gentamicin (GEN), Imipenem (IMP), Levofloxacin (LEV), Lincomycin (LIN), Meropenem (MER), Minocycline (MIN), Netilmicin (NET), Norfloxacin (NOR), Ofloxacin (OFX), Penicillin (PEN), Tetracycline (TET), Tobramycin (TOB).

**Fig. 6:**
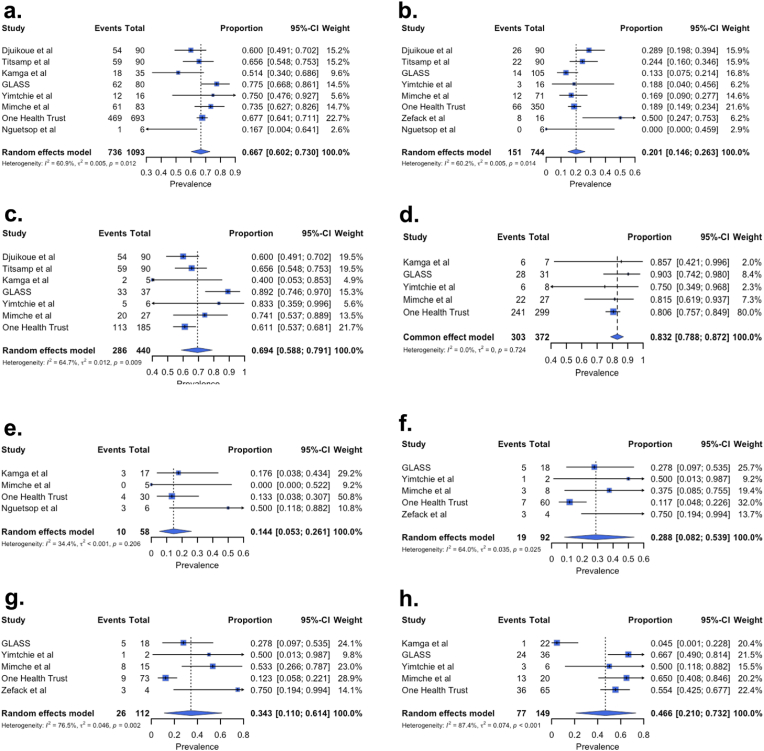
Forest plots showing pooled prevalence of resistance phenotypes. **(a)** Third-generation cephalosporin-resistant *Enterobacterales*. **(b)** Carbapenem-resistant *Enterobacterales*; **(c)** Third-generation cephalosporin-resistant *Escherichia coli*, including 64 phenotypically confirmed extended-spectrum β-lactamase (ESBL) producers out of 186 tested [11], [13], [19]; **(d)** Third-generation cephalosporin-resistant *Klebsiella* species, including three phenotypically confirmed ESBL producers out of eight tested [3]. **(e)** Fluoroquinolone-resistant *Salmonella* species; **(f)** Carbapenem-resistant *Acinetobacter* species; **(g)** Carbapenem-resistant non-fermenting Gram-negative bacilli; (**h)** Methicillin-resistant *Staphylococcus aureus*.

This study reports only the pooled AMR rates of the most represented bacteria and antibiotics tested against at least 20 isolates, although below the minimum of 30 isolates suggested by the CLSI [32], this cut-off is chosen to not miss out clinically relevant antibiotics considering the available data. Overall, *E. coli* displayed elevated resistance rates to ampicillin 98.0 % (92.1–100.0 %, n = 68, k = 02), amoxicillin 83.8 % (65.1–96.9 %, n = 295, k = 06), amoxicillin + clavulanic acid 77.2 % (55.3–93.9 %, n = 375, k = 07), and to cotrimoxazole 76.8 % (57.3–92.3 %, n = 272, k = 07) (Fig. 5a–S10-11). Additionally, the resistance to third-generation cephalosporins was substantial 69.4 % (58.8–79.1 %, n = 440, k = 07) (Fig. 6c), as well as to cefepime (fourth-generation cephalosporin) 58.1 % (46.9–68.9 %, n = 218, k = 05), while moderate resistance of 27.5 % (8.7–50.8 %, n = 218, k = 06) was observed against cefoxitin (cephamycin). These isolates were therefore the least resistant to carbapenems, including imipenem 9.9 % (4.1–17.3 %, n = 150, k = 05), meropenem 19.0 % (9.8–30.1 %, n = 245, k = 05) and ertapenem 23.2 % (16.1–31.1 %, n = 137, k = 04). The AMR profile of *Klebsiella* species revealed an alarming resistance to third-generation cephalosporins of 83.2 % (78.8–87.2 %, n = 372, k = 05), cefepime 75.3 % (62.0–86.8 %, n = 52, k = 03) and cotrimoxazole 79.0 % (49.7–98.3 %, n = 66, k = 04). Among aminoglycosides, the resistance rate was least against amikacin 28.3 % (22.5–34.3 %, n = 264, k = 04), compared to gentamicin, netilmicin and tobramycin, whose resistance rates were twice as high. As observed in *E. coli*, *Klebsiella* isolates were less resistant to carbapenems, particularly to ertapenem 2.4 % (0–11.4 %, n = 46, k = 03) (Fig. 5, Fig. 6d and S10, S11). Resistance rates of *Salmonella* species were around 10 % for most of the tested antibiotics except for cotrimoxazole 49.8 % (19.4–80.2 %, n = 27, k = 03), amoxicillin + clavulanic acid 39.2 % (16.4–64.3 %, n = 20, k = 02), ofloxacin 22.7 % (0–71.6 %, n = 20, k = 02), and ceftriaxone 18.7 % (6.0–35.0 %, n = 35, k = 03). The least resistance rates were registered to imipenem 2.3 % (0–13.8 %, n = 36, k = 04) and cefixime 4.5 % (0–14.1 %, n = 53, k = 03) (Fig. 5d–S10-11). Fluoroquinolones-resistant *Salmonella* was 14.4 % (5.3–26.1 %, n = 58, k = 04) (Fig. 6e). We also estimated the resistance of *Enterobacterales* against third-generation cephalosporins and carbapenems and found resistance rates of 66.7 % (60.2–73.0 %, n = 1093, k = 08) and 20.1 % (14.6–26.3 %, n = 744, k = 08) respectively (Fig. 6a and b).

In *Acinetobacter* species, resistance rates against third- and fourth-generation cephalosporins ranged from 68.0 % to 89.5 %, with a 95 % CI that reached 100 %, and resistance were less than 20 % for ciprofloxacin, amikacin and ofloxacin (Fig. 5c). Carbapenems resistance rate was 28.8 % (8.2–53.9 %, n = 92, k = 05) while for all non-fermenting Gram-negative bacilli including but not limited to *Pseudomonas* species, *Burkholderia cepacia*, *Shingomonas paucimobilis*, *Shingobacterium multivorum*, *Achromobacter xylosoxidans* and *Stenotrophomonas maltophilia* was estimated at 34.3 % (11.0–61.4 %, n = 112, k = 05) (Fig. 6f and g).

Staphylococci globally maintained elevated resistance rates against all tested antibiotics, with gentamicin showing the lowest rate of 30.0 % in *Staphylococcus aureus*. Methicillin-resistant *Staphylococcus aureus* was reported in 46.6 % (21.0–73.2 %, n = 149, k = 05) of cases, and similarly, 46.0 % (34.6–57.5 %, n = 78, k = 03) of coagulase-negative staphylococci were cefoxitin-resistant (Fig. 5, Fig. 6h and S10-11). The resistance rate of Staphylococci to vancomycin, although an important resistance phenotype, was not reported in this study because almost all studies that tested this antibiotic used the Kirby-Bauer method, while both CA-SFM and CLSI guidelines recommend the determination of the MIC instead.

This study reveals several issues in microbiology data and noticeable reporting gaps, which may significantly hamper analysis, particularly that of AMR. Thus, these results should be cautiously interpreted. The study also highlights the need for improved laboratory quality management and assessment to make local, national, and global AMR burden estimates more robust.

## 4 Discussion

This review aimed to determine the pooled prevalence of bacterial BSIs and the AMR rates of isolated bacteria in Cameroon. The ten studies included in the meta-analysis of BSI investigated 5680 blood cultures, with a pooled prevalence of 25.2 % and 17.2 % when potential contaminants were removed. Although the high heterogeneity observed across studies may have significantly contributed to the publication bias, a reduced prevalence of 14.8 % was found when potential biases were adjusted through the fill and trim method. The subgroup analysis revealed that the study population age and hospital settings where the studies were conducted explained part of the heterogeneity. Another potential source of heterogeneity could be the origin of the BSI—there was no clear specification in the included studies as to whether the BSI was community- or hospital-acquired, and we were therefore unable to split the data into subgroups without introducing bias. Similarly, the fact that most studies may have used a single blood culture set to investigate BSI has made it more challenging to properly handle contaminants, which would probably have inflated BSI prevalence in some studies and consequently the heterogeneity across studies. Despite the robustness of the BSI pooled prevalence in this review and the substantial number of blood cultures included, it may be overestimated and misleading in the real context across the country because data from most regions are missing, and the available ones are almost from the same hospitals.

The leading bacteria causing BSI included *Escherichia coli*, *Klebsiella* species, and *Staphylococcus aureus*. Studies conducted in several low-and middle-income countries have documented the strong implications of these bacteria in BSIs, especially in hospital settings [9,33]. Results of a systematic review and meta-analysis in Africa carried out by Reddy et al. have highlighted the strong implication of *Salmonella* species among the *Enterobacterales* in the community-acquired BSIs, instead [8]. In Cameroon, a study conducted at Yaoundé University Teaching Hospital (YUTH) by Gonsu et al. reported high nasal, faecal and hand carriage of multidrug-resistant *Staphylococcus aureus*, *Klebsiella pneumoniae*, *Escherichia coli* and *Enterobacter cloacae* among healthcare workers, and concluded that they were important reservoirs of resistant bacteria [34]. More interestingly, in another study conducted in the major tertiary category hospitals of Yaoundé, including YUTH, 54 % of the included physicians disagreed that poor hand hygiene is a cause for the spread of antibiotic-resistant bacteria [35]. Thus, attitudes and practices of medical personnel, such as low hygiene and safety measures, may significantly contribute to the spread of resistant bacteria to patients and suggest that a greater share of BSIs reported in this review are of nosocomial origin. Although almost all studies documented the use of acceptable identification techniques and meta-analysis included up to 1000 isolates for *E. coli* and about 800 isolates for other bacteria, some studies did not present the full list of isolated bacteria [11,13,17]. This reporting gap, combined with the use of the manual blood culture technique in most studies, which may limit the isolation of some bacteria, means that the spectrum of BSI-causing bacteria in this review may not represent the full picture.

Our findings revealed substantial resistance rates of isolated bacteria to commonly used antibiotics in the clinic, particularly the extended-spectrum cephalosporins in *E. coli*, *Klebsiella* species and *Acinetobacter* species. In both *Staphylococcus aureus* and CoNS, the resistance rate was overall elevated against all antibiotics. Additionally, the contribution of bacterial priority pathogens as defined by the WHO [36], including carbapenem-resistant *Acinetobacter* (28.8 %), third-generation cephalosporin-resistant *Enterobacterales* (66.7 %), carbapenem-resistant *Enterobacterales* (20.1 %), fluoroquinolone-resistant *Salmonella* (14.4 %), and methicillin-resistant *Staphylococcus aureus* (46.6 %) was significant. Many studies conducted in Cameroon recorded the overuse and misuse of frontline antibiotics in hospital and community settings [35,37]. Moreover, a study by Domche et al. also highlighted irrational prescription of antibiotics and low level of knowledge on antibiotic use of medical doctors from major tertiary hospitals included in the study [35]. All these practices and gaps in antibiotic knowledge may have significantly contributed to the emergence and spread of resistant bacteria in the hospital environment and among patients through the selection pressure exerted by antibiotics. Although the AMR data of studies conducted by international organisations [28,29] were included in the analysis of AMR rates, we think that these results may be a bit overestimated and should therefore be used with caution. Some methodological gaps and a lack of compliance with international guidelines for AST were noticed in many included studies. For example, a few studies reported the use of the control strains to validate the quality of antibiotic discs, many studies tested antibiotics against a bacterium which possesses intrinsic resistance to the antibiotic (e.g., penicillin against *Klebsiella* species) or used an unrecommended AST technique for some antibiotics (e.g., the use of disc diffusion method to test the susceptibility of staphylococci against vancomycin). Discrepancies in resistance rates among the tested carbapenems (imipenem, meropenem, and ertapenem) in *E. coli*, *Klebsiella* species, and *Acinetobacter* could illustrate the problem— it is likely due to the quality of the disc or other laboratory errors rather than the actual resistance.

## 5 Limitations

The statements and conclusions in this review cannot be generalised to Cameroon, as the included studies investigated only two of the ten regions and included data from a few tertiary hospitals. The lack of reporting whether the bloodstream infection was of community or hospital-acquired origin may have significantly contributed to the high heterogeneity and misleading prevalence/AMR estimates. The AMR rates reported in this review may be overestimated due to several gaps and inconsistencies identified in laboratory work, and should not therefore be considered in a strict sense.

## 6 Conclusion

This review provides comprehensive data regarding bacterial BSI prevalence and AMR epidemiology in Cameroon. The prevalence of bacterial bloodstream infections in Cameroon is much higher than reported in some studies conducted across Africa, thus implying a likely more devastating burden. *Escherichia coli*, *Klebsiella* species, and *Staphylococcus aureus* are the most predominant isolated pathogens. Gram-negative bacilli causing BSIs, except *Salmonella* species, displayed overall important resistance against antibiotics used in the first-line treatment of BSIs in Cameroon, particularly β-lactams. Both *Staphylococcus aureus* and coagulase-negative staphylococci consistently maintained elevated resistance rates to all tested antibiotics. The WHO bacterial priority pathogens reported in this review occupied a greater proportion of the overall BSI-driven bacteria. Further epidemiological studies are needed to enhance our understanding and to enable policymakers and healthcare stakeholders to develop more targeted strategies for the prevention, management, and treatment of bloodstream infections in Cameroon. More efforts are also needed across the country to empower the laboratories’ capacity in microbiological data analysis and AMR surveillance, particularly in compliance with international guidelines.

## CRediT authorship contribution statement

**Moise Matakone:** Writing – review & editing, Writing – original draft, Visualization, Formal analysis, Data curation, Conceptualization. **Sen Claudine Henriette Ngomtcho:** Writing – review & editing, Writing – original draft, Validation, Supervision. **Patrice Landry Koudoum:** Writing – review & editing, Writing – original draft, Data curation. **Isaac Dah:** Writing – review & editing, Validation, Data curation. **Ravalona Jessica Zemtsa:** Writing – review & editing, Data curation. **Michel Noubom:** Writing – review & editing, Validation, Supervision.

## Availability of data and materials

All the data generated and analysed during the current study are included in the manuscript and supplementary materials.

## Funding

This research did not receive any specific grant from funding agencies in the public, commercial, or not-for-profit sectors.

## Declaration of competing interest

The authors declare that they have no conflicts of interest.
