# Supplementary material for: Bloodstream infections in Cameroon: a systematic review and meta-analysis
Source: New Microbes New Infect. 2025 Oct 14;68:101654. doi: 10.1016/j.nmni.2025.101654 (PMC12554137; doi:10.1016/j.nmni.2025.101654)
Supplement: Multimedia component 1 [file mmc1.docx]

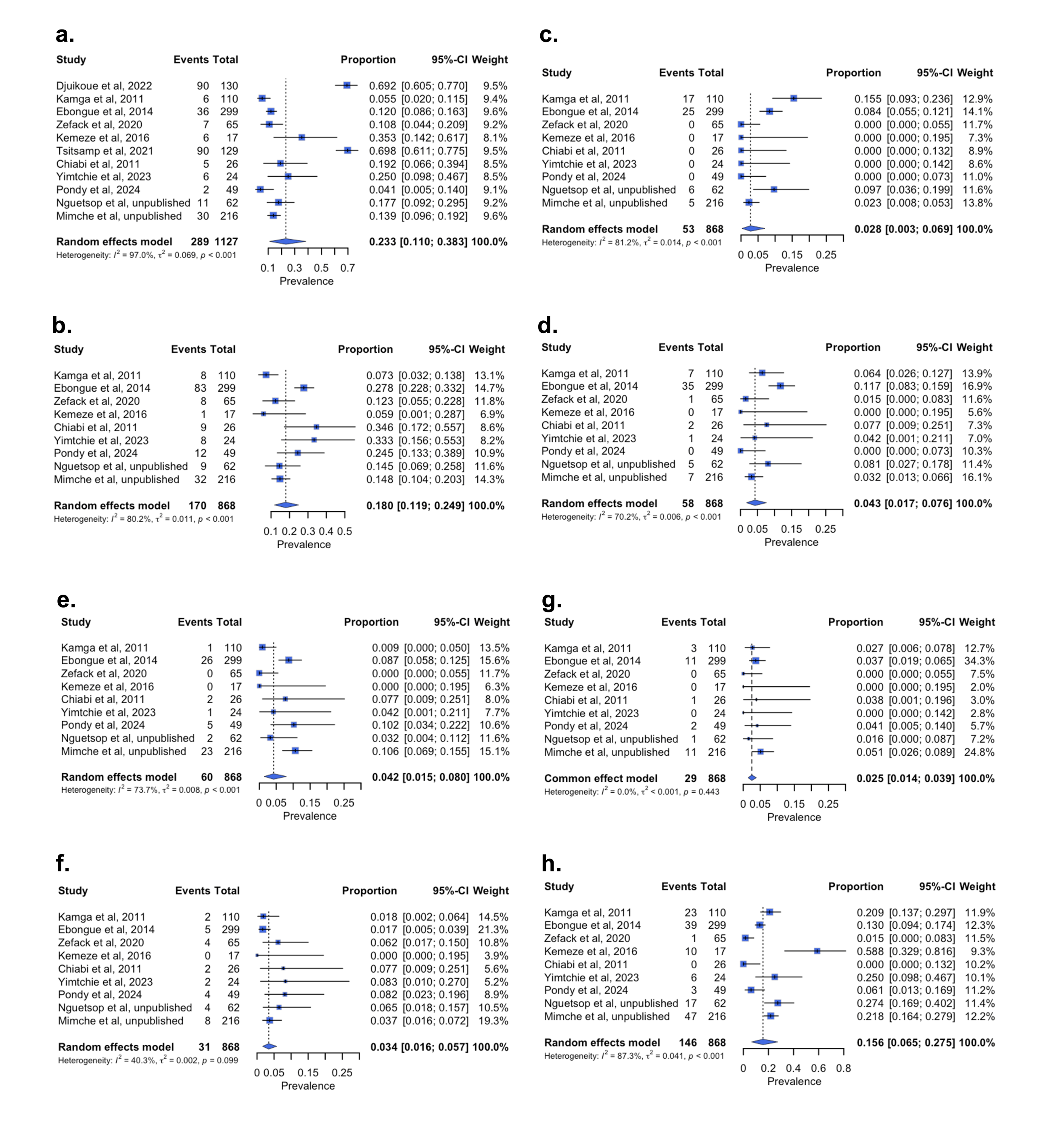


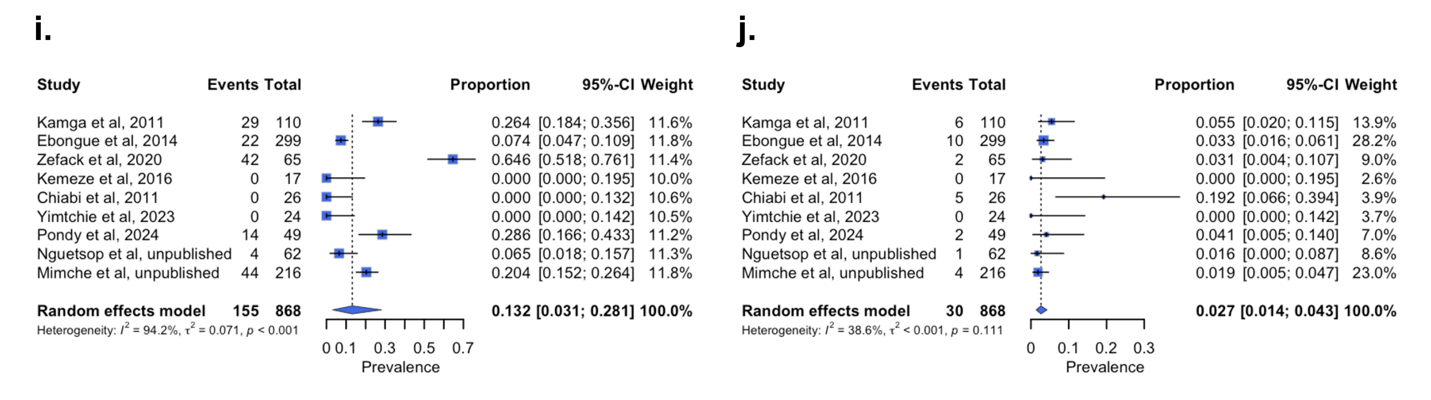


**Supplementary Fig.** **1**. **(a)** *Escherichia coli*; **(b)** *Klebsiella* species among which 138 were *Klebsiella pneumoniae*; **(c)** *Salmonella* species among which 11 were *Salmonella* Typhi; **(d)** *Enterobacter* species; **(e)** Other *Enterobacterales* includes *Citrobacter* species (n = 08), *Yersinia enterocolitica* (n = 02), *Proteus* species (n = 07), *Serratia* species (n = 03), *Providencia* species (n = 07), *Morganella morganii* (n = 01), *Pantoea* species (n = 04), *Kluyvera cryocrescens* (n = 01), *Escherichia hermannii* (n = 01), unspecified *Enterobacterales* (n = 26); **(f)** *Acinetobacter* species, among which 11 were *Acinetobacter baumannii*; **(g)** *Pseudomonas* species, among which 18 were *Pseudomonas aeruginosa*; **(h)** *Staphylococcus aureus*; **(i)** Coagulase negative staphylococci, including *Staphylococcus epidermidis* (n = 47), *Staphylococcus saprophyticus* (n = 19), *Staphylococcus haemolyticus* (n = 12), *Staphylococcus simulans* (n = 01), *Staphylococcus gallinarum* (n = 01), *Staphylococcus xyloxus* (n = 02), *Staphylococcus hominis* (n = 03), *Staphylococcus lugdunensis* (n = 01), *Staphylococcus cohnii* (n = 01), *Staphylococcus* species (n = 02) and unspecified CoNS (n = 66); **(j)** *Streptococcus* species.
